# Sinking towards destiny: High throughput measurement of phytoplankton sinking rates through time-resolved fluorescence plate spectroscopy

**DOI:** 10.1371/journal.pone.0185166

**Published:** 2017-10-03

**Authors:** Catherine C. Bannon, Douglas A. Campbell

**Affiliations:** Biology Department, Mount Allison University, Sackville, NB, Canada; University of Hyderabad School of Life Sciences, INDIA

## Abstract

Diatoms are marine primary producers that sink in part due to the density of their silica frustules. Sinking of these phytoplankters is crucial for both the biological pump that sequesters carbon to the deep ocean and for the life strategy of the organism. Sinking rates have been previously measured through settling columns, or with fluorimeters or video microscopy arranged perpendicularly to the direction of sinking. These side-view techniques require large volumes of culture, specialized equipment and are difficult to scale up to multiple simultaneous measures for screening. We established a method for parallel, large scale analysis of multiple phytoplankton sinking rates through top-view monitoring of chlorophyll *a* fluorescence in microtitre well plates. We verified the method through experimental analysis of known factors that influence sinking rates, including exponential versus stationary growth phase in species of different cell sizes; *Thalassiosira pseudonana* CCMP1335, chain-forming *Skeletonema marinoi* RO5A and *Coscinodiscus radiatus* CCMP312. We fit decay curves to an algebraic transform of the decrease in fluorescence signal as cells sank away from the fluorometer detector, and then used minimal mechanistic assumptions to extract a sinking rate (m d^-1^) using an RStudio script, SinkWORX. We thereby detected significant differences in sinking rates as larger diatom cells sank faster than smaller cells, and cultures in stationary phase sank faster than those in exponential phase. Our sinking rate estimates accord well with literature values from previously established methods. This well plate-based method can operate as a high throughput integrative phenotypic screen for factors that influence sinking rates including macromolecular allocations, nutrient availability or uptake rates, chain-length or cell size, degree of silification and progression through growth stages. Alternately the approach can be used to phenomically screen libraries of mutants.

## Introduction

Diatoms (Class: *Bacillariophyta*) are algae that evolved from a tertiary endosymbiosis event between a heterokont and and a red alga (Archibald and Keeling, 2002). They are characterized by their siliceous cell walls, called frustules, that vary in shape and size and which are used for taxonomic assignments. These unicellular organisms are the most abundant and ecologically successful group of eukaryotic phytoplankton [1]. In parallel with their genetic diversity, they occupy aquatic habitats that present wide ranges of environmental conditions [2]. Diatoms play a pivotal role in maintaining global climate through their role the carbon cycle, by contributing up to 35% of primary productivity from oligotrophic oceans and up to 75% from coastal waters [3]. Diatoms, more than any other phytoplankton, export carbon from surface areas through sinking into the deep ocean after photosynthetic carbon dioxide fixation [4]. The large cell sizes of some strains, heavy siliceous cell walls, and chain forming character of some taxa, promote this sinking. They are collectively responsible for 40% of the CO_2_ flux to the deep ocean [5]. Sinking of diatoms introduces organic carbon from the photic zone into the deeper ocean food web or to the bottom of the ocean. This process sequesters carbon from the atmosphere into the deep ocean, and modulates oceanic energy and nutrient cycling. Sinking rate is likely second only to growth rate as a key ecophysiological parameter for the interactions of phytoplankton with their environment and their trophic connections to the wider community.

Historically large diatom sinking events were associated with mass death of a community [6]. This idea was challenged by the idea of diatoms ‘seeded’ out of blooms to deep waters or sediments, to increase the probability of eventually reaching more favourable environments [7–10]. There is an intricate balance between light energy that drives photosynthesis and the nutrient uptake of components essential for cellular growth and division [11]. The position of a phytoplankter in the water column dictates their access to each of these key requirements, thereby controlling their life history strategy [10].

Cell size has a crucial influence on how fast a phytoplankter will sink. All other factors being equal, a larger cell will sink faster than a smaller cell. This theory also extends to chain-forming phytoplankters as a longer chain will sink faster than a shorter chain of the same phytoplankter. These patterns result from Stoke’s Equation:

$$v_{s}=2gr^{2}(\rho^{\prime }-\rho )(9\eta \phi {)}^{-1}$$ (1)

where *v*_*s*_ is sinking velocity (or velocity of floating if cellular density is less than sea water) [m s^-1^]; *g* is the acceleration of gravity [9.8 m s^-2^]; *r* is the radius of a spherical approximation of the sinking particle [m]; *pʹ* is the particle density [kg m^-3^]; *p* is the density of the water; ***η*** is the viscosity of water [kg m^-1^ s^-1^]; and *ϕ* is the form resistance, which states how slowly a particle sinks compared to a sphere of equal volume [12]. The sinking rate is thus proportional to the square of the radius of the cell [12], whereas sinking rate is only linearly proportional to the density difference between the cell and the media. Diatoms can actively control their buoyancy through cellular regulation of osmolytes that can even lead to positive buoyancy [13]. The larger diatom *Coscinodiscus wailesii* indeed has the capacity to control buoyancy within seconds in a mechanism under direct control of the diatom metabolism [14]. This ability of large diatoms to rapidly regulate passage through water can afford an enhanced nutrient flux by refreshing the composition of the cellular boundary layer. Diatom sinking rates also vary throughout the organism life cycle. Growth rates within a species are inversely correlated to the species sinking rates [15]. Non-growing cultures will sink faster than growing cultures [16]. Phytoplankton strategically place themselves higher in the water column to obtain more light energy during the rapid cellular division of exponential phase. Once nutrient depletion limits growth the culture of diatoms will reach carrying capacity for that specific environment, and cells will begin to sink to explore and exploit new surroundings for depleted nutrients, possibly by controlling their intracellular carbohydrate and protein ratios [11], or more directly through control of ion pumps. [13,14].

Different methodologies and equipment have been developed to analyze sinking rates of phytoplankters and the factors influencing sinking rates. Settling columns (SETCOL) estimate sinking rate through the change over a given time of vertical distribution of biomass that is initially uniformly suspended in a column of known height [17]. This approach uses large volumes of sample and the process of sedimentation can be time consuming. A newer approach [14] used a Nikon 7100 DSLR camera with a macro lens and high pixel resolution to track the movement of diatoms in a water column. The cells are repeatedly captured in images to resolve the time taken to move a known distance. This technique for high resolution analyses of single cells uncovered their capacity for rapid regulation of buoyancy. Fluorimeters can eliminate the need to visually count cells and has generally greater sensitivity than video imaging techniques. Using fluorescence as a proxy for local cellular concentration the sinking rate of phytoplankters has been been assessed [16] by tracking the change in fluorescence as cells settle past a detector arranged perpendicularly to the direction of sinking. Such techniques again require extensive culture and time, with single channel side-view equipment that is difficult to scale up for phenotypic screens or high through put analyses of mutant collections. Thus, there is a need for a technique for high throughput determination of sinking rates of phytoplankters using only small sample volumes, to compare multiple strains or mutant lines responding to ranges of environmental factors. We therefore developed a method to monitor sinking rates of phytoplankton through top view of fluorescence of chlorophyll *a* in microtitre-well plates, compatible with high throughput or robotized phenomic screening experiments of mutant collections or taxa responses to environmental variables.

## Materials and methods

### Diatoms and culture conditions

Stock cultures were maintained at 16°C under 60 μmol m^-2^ s^-1^ fluorescent light on a diurnal cycle (12:12, light:dark) in an Percival incubator. Cultures of *Thalassiosira pseudonana* CCMP1335 (Panel a in S1 Fig) and *Coscinodiscus radiatus* CCMP312 (Panel b in S1 Fig)were diluted 1:25 every 10–14 days into new f/2 media. Wild type *Skeletonema marinoi* RO5A cultures (Panel c in S1 Fig) were diluted every 10 days into fresh enriched artificial sea water medium (EASW) at a 1:25 dilution. Salinity of EASW was tested and corrected to 26 practical salinity units (37 mS cm^-1^) using a refractometer while pH was adjusted to 8.1 (±0.5) using 0.1 M HCl solution. For experimental purposes, all cultures were shifted to 120 μmol m^-2^ s^-1^ fluorescent light within an incubator to produce near light-saturated growth rates.

### Tracking growth with spectrofluorometer

Culture growth was read daily in a 96 well plate using a fluorescence plate spectrofluorometer (Molecular Devices SpectraMax Gemini EM, Sunnyville, California). The chlorophyll of cultures was excited by a modulated flash lamp at 435 nm and the resulting cumulative chlorophyll fluorescence emission provoked by a train of 6 flashlets was read at 680 nm[18] for all taxa. The Molecular Devices instrument is capable of driving chlorophyll fluorescence induction (D. Campbell, unpub.) by varying the number of flashlets applied per read, but within a given growth curve all measures were captured with the same excitation/emission settings, to achieve approximately equivalent fluorescence induction to F_O_, for each measure, with little influence on the growth results through changes in fluorescence yield. Growth curves for each culture were then constructed in R Studio [19] by importing specifically named files of the plate spectrofluorometer output for processing.

Exponential growth rate of cultures was estimated using a Gompertz growth rate formula:

$${RFU}_{t}=A*\;exp[-\exp\left(\frac{\mu *e}{A}(\lambda -t)+1\right)]$$ (2)

where RFU_t_ is relative fluorescent unit at a point of time and A is ln(RFU_max_/RFU_min_). After curve fitting the actual estimate of RFU_max_ can then be generated as anti-ln(RFU_max_/RFU_min_) x RFU_min_. λ is lag phase, μ is the exponential growth rate, and e is the elapsed time [20]. Cultures were considered to be in stationary phase after 3 days of stable RFU.

### Sinking assay measurement protocol

Sinking rate assays were performed by monitoring chlorophyll *a* fluorescence in the same plate spectrofluorometer (Molecular Devices SpectraMax Gemini EM, Sunnyvale, California). To begin an experiment for a 96 well-plate, 300 μL of culture was carefully pipetted into a well containing a parylene-coated stir bar (VP 711D-1, V&P Scientific, INC). For experiments that required 24 well-plates 2.8 mL of cultures were used in a well along with a parylene-coated stir bar. The culture sample depth was 3.8 mm in both 96 and 24 well plates, with the difference in volume caused by the difference in diameter, not the depth, of the well. The addition of the stir bar aided the thorough re-suspension of cells in all rows and column of well plates and thereby helped to minimize variation generated by the unequal re-suspension of diatoms in the well column during shaking of the well plate.

**Fig 1:**
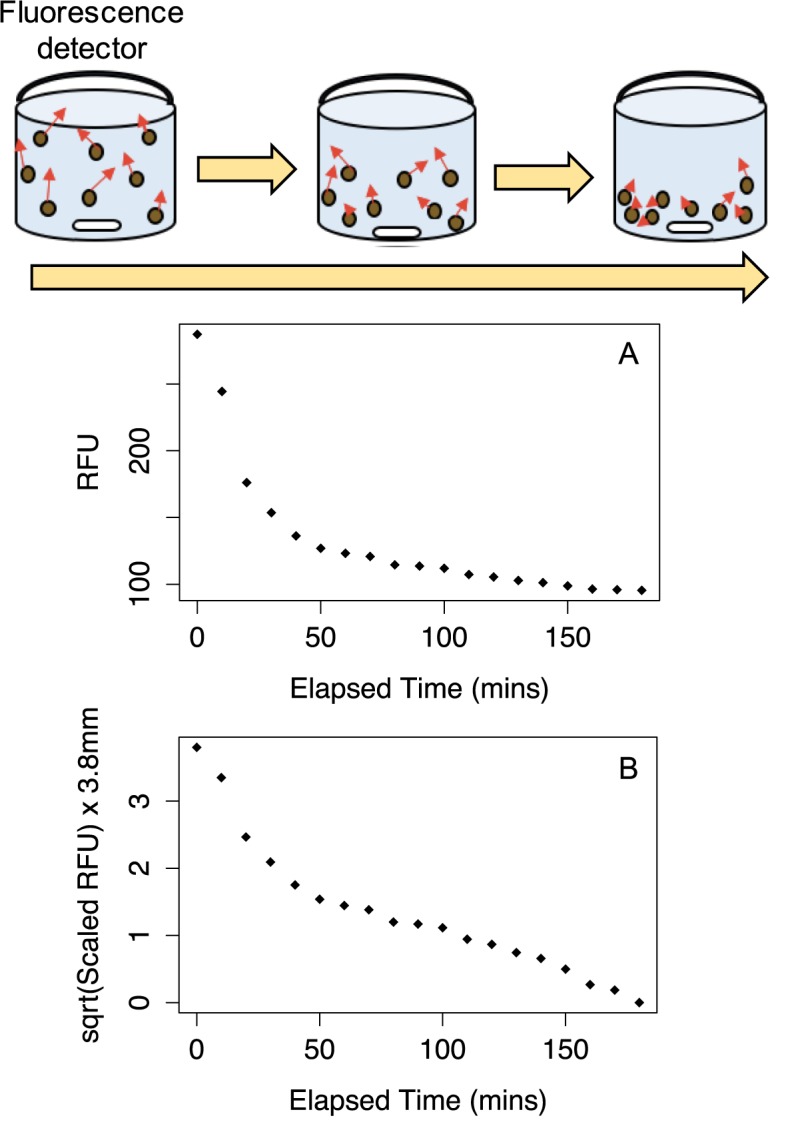
Sinking assay schematic and data. One well is depicted at different time points, left to right, during the sinking assay. The well contains phytoplankton culture with isotropic fluorescence emission symbolized by red arrows, a stir bar and the fluorescent detector at the top. The isotropic characteristic of emitted fluorescence permits the monitoring of phytoplankton sinking. **(A)** Relative Fluorescence (RFU) data plotted *versus* Elapsed Time. **(B)** Square root of scaled Relative Fluorescence data, multiplied by 3.8 mm sinking depth for well-plate.

After acclimation (16°C, 120 μmol m^-2^ s^-1^) for a minimum of 10 minutes, well-plates were mixed on a Cooke Microtiter Micro Mixer for 30 seconds and then placed on the stage of plate spectrofluorometer. Fluorescence (excitation: 445 nm; emission: 680 nm), with the same settings used to measure culture growth, was repeatedly monitored from the top of the well-plate during a determined time course. The distance from the photodioded fluorescence detector to the top of the culture (~2 mm) was consistent between the 96 and 24 well plates and fluorescence was measured from the central region of each culture well. After the sinking assay time course, the spectrophotometer (Molecular Devices, SpectraWorks) generated.txt file was exported to a Dropbox directory for processing by the SinkWORX script (https://www.dropbox.com/sh/w03c2nt97rjk990/AACyg_nJrl75ztT9s6A89zT-a?dl=0) (Fig 1).

The largest relative fluorescent (RFU) will be received by the detector when Elapsed Time is 0, when the cells are initially evenly suspended. As the cells sink, the likelihood of the isotropic fluorescent emission reaching the detector decreases following the inverse square law as cells move away from the detector, until the RFU hits a plateau once cells in the well were resting at the bottom and giving the same RFU at the detector with each successive read.

Before and after the time course, during initial sinking assay trials, we also measured fluorescence from the bottom of the clear plates to confirm proportional but opposing changes in fluorescence due to sinking when measured from the top or the bottom (data not presented).

### Proof of concept experiments

Cell size and growth phase influence sinking rate of diatoms [12,21]. These factors were studied to examine the sensitivity of our method to detect expected changes in sinking rates. Three taxa of different cell or filament length were grown in near-saturating light while daily growth measurements were taken. When cultures reached exponential and then again during stationary phase, sinking assays were performed prior to diatom fixing for cell and filament sizing.

### Cell and filament measurements

For cell and filament measurements diatoms were fixed by pipetting 200 μL culture from each of three replicate well plate wells into a 2 mL micro centrifuge tube containing 1.8 mL of 2.5% glutaraldyhyde in artificial sea water, for a final concentration of 0.225% glutaraldyhyde. Microtubes were then placed in 4°C refrigerator for storage until cell measurements were taken. From each tube, 40 μL of fixed diatoms in medium was pipetted onto glass slide and then covered with a cover slip. Cell diameter and chain length were measured using a Zeiss Axio Imager A1 microscope. Cells were viewed under 40X objective using ToupView.64 software. Cell and filament length were measured from 10 arbitrarily selected cells or filaments, measured with a straight line from edge to edge of each cell or chain. Data was then exported as a.csv for further analyses.

### Data conversions

The conversion, analysis and comparison of data produced by the sinking assay was performed in R-Studio Software Version 0.99.486 using our SinkWORX data analysis pipeline. The package segmented [22] was used for break point analysis and ggplot2 [23] for figure plotting. The annotated SinkWORX R-script directory with example data is provided as S1 Script.

Meta-data on the culture sample in each well in a 96 well-plate used for sinking assay was first entered manually using Microsoft Excel in a SinkingCulture_Catalog.csv data sheet. Raw data produced from each sinking assay was imported from the specifically named Molecular Devices export file and converted in R-scripts to be plotted as RFU from each plate well against elapsed time (minutes), using the meta-data on the well content to tag each sinking profile.

We then scaled RFU:

$$Scaled\;{RFU}_{t}=\frac{{RFU}_{t}-min(RFU)}{\max\left(RFU\right)-\min\left(RFU\right)}$$ (3)

*RFU*_*t*_ is the relative fluorescent signal of the individual time point on which scaling is being performed. The initial even suspension of cells in each well thus generated a scaled RFU of 1 while the final RFU plateau at min (RFU) scaled to 0 once all cells reach the bottom 3.8 mm below the well surface in our system (Fig 1).

Scaling was performed to minimize the variation in the raw fluorescence data that was produced from arbitrary differences in initial cell suspension density among wells. In particular samples from stationary phase cultures typically had much higher cell densities than cultures from exponential phase.

As cells sink towards the bottom of the well, moving away from the detector, we reasoned that scaled RFU initially declines:

$$Scaled\;{RFU}_{t}=\frac{1}{{\left(1+d_{t}\right)}^{2}}$$ (4)

where d_t_ is the distance sunk downwards by a cell starting from the top of well over elapsed time t. Empirical and theoretical estimates show that the bulk of our fluorescence signal results from cells starting at the top of the well because:

1. Cells initially lower in the well receive less excitation light because they are initially farther from the emitter and they are screened by cells above them.
2. Cells initially lower in the well therefore emit less fluorescence light, which in turn is attenuated by re-absorption by cells above them, so less fluorescence reaches the detector from cells low in well.
3. Therefore the received fluorescence signal is weighted towards cells at the top of the well and this weighting becomes progressively stronger as the cell suspension density increases, either through a higher starting cell suspension density or as the cells collapse into a dense layer at the bottom of the well. A more sophisticated analyses could, if needed, be derived from work on mudflat diatoms where steep vertical attenuation of light influences measured fluorescence [24,25].

Our goal is to extract a sinking rate for the phytoplankton cells in suspension.

We reasoned that as cells sink:

$$d_{t}=t\;(min)\times sinking\;rate\;({min}^{-1})$$ (5)

where d_t_ is the distance between the emitting cell and the detector at elapsed time t, and s (min^-1^) is the scaled sinking rate.

Combining the two equations:

$${Scaled\;RFU}_{t}=\frac{1}{{\left(1+(t*s)\right)}^{2}}$$ (6)

Then

$$\sqrt{{Scaled\;RFU}_{t}}=\frac{1}{\left(1+(t*s)\right)}$$ (7)

which, by a geometric series, approximates to

$$\sqrt{{Scaled\;RFU}_{t}}∼1-(t\times s)$$ (8)

This gives us a linear formulation to estimate scaled sinking rate. For comparison with literature values of sinking rates the scaled sinking rate was multiplied by the actual distance travelled, the depth of the culture in the well which in our experiments was 3.8 mm. This value was then converted to meters d^-1^. It was assumed that cells were not affected compositionally by the experiment and that fluorescence is in fact proportional to concentration of cells, at least over the forty minute to three-hour time scale of the sinking experiment.

Some cultures showed an initial increase of measured fluorescence during the first thirty minutes of the sinking assay. This rise in fluorescence could be attributed to the slow relaxation of a phase of the organism's non-photochemical quenching mechanism [26] that resulted from the decrease in light intensity throughout the preliminary steps of the sinking assay protocol compared to the preceding growth light level. Non-photochemical quenching is a mechanism to dissipate excess light-energy in the form of heat to protect the photosynthetic complexes [26–28]. The relaxation of this mechanism would increase fluorescence emission from cultures as they re-allocate excitation from heat dissipation pathways back to photosynthetic processes.

The sinking of diatoms in the first thirty minutes of assay can thus be masked by the rising fluorescence signal of NPQ relaxation [26]. For analytical purposes in this study we plotted the square root of scaled RFU versus elapsed time and fit the data with a segmented linear regression to separate the initial rise phase from the sinking phase. We then extrapolated back to the y-intercept and rescaled this y-intercept to one. Such detection and correction of NPQ responses will be necessary for accurate assessment of sinking rate, since the amplitudes and relaxation rates of measured NPQ vary with taxa and with growth conditions [26,29], with complex effects upon fluorescence yields[30]. There is no single pre-measurement incubation treatment or period that would guarantee escape from such variations across diverse phytoplankton taxa. Instead we include the data to demonstrate the need for caution when attributing changes in fluorescence signals in terms of cell sinking.

### Statistical analysis

R-studio software was used to statistically analyze triplicated sinking rates using two 2-way ANOVA with two fixed factors (Growth Phase, Species) and one dependent variable extracted from segmented regression of scaled data. (Sinking Rate of First and Second Phase, meter day^-1^). Packages car [31], psych [32] and sciplot [33] were used for statistical analysis. Breakpoint was manually fit for one well of *C*. *radiatus* in exponential phase in order to obtain the first phase sinking rate. It was calculated as the average of two breakpoints fit by break point analysis in other wells of triplicate. A logarithm transformation of first phase sinking rate was performed to meet the assumption of normality required for the ANOVA[34]. Interaction between Phase and Species was not significant (p-value = 0.695) therefore a Tukey’s honest significance test was performed to analyze significance between levels. Data and data transformations supporting Figs 2 to 5 are given in S1 Data–S11 Data.

For statistics involving the study of well plate effect on sinking rates, we performed one-way ANOVAs on the sinking rates produced by each well in triplicate and the independent variable, Well Plate (24 well-plate, 96 well-plate). Data and statistical analyses for well-plate effect and for proof of concept experiments are given in S1 Statistics.

## Results and discussion

We aimed to establish a technique for the differential analyses of phytoplankton sinking rates through plate spectroscopy, compatible with high throughput screening. Factors already known to influence sinking rates in diatoms were cell size and phase of growth. As such, the growth of three different diatoms that ranged in size and morphology were tracked daily and then sampled for the sinking assay in triplicates during their exponential phase and then again, for two species, in stationary phase. Two unicellular centric diatoms, one relatively small (*Thalassiosira pseudonana* CCMP1335*)* and one relatively large (*Coscinodiscus radiatus* CCMP312*)*, were studied along with a chain-forming coastal diatom whose chains represented an intermediate diatom particle size (*Skeletonema marinoi RO5A)*.

### Sinking rate analysis of chain forming diatom

#### Sinking rate analysis of *Skeletonema marinoi* in exponential and stationary growth phase

**Fig 2:**
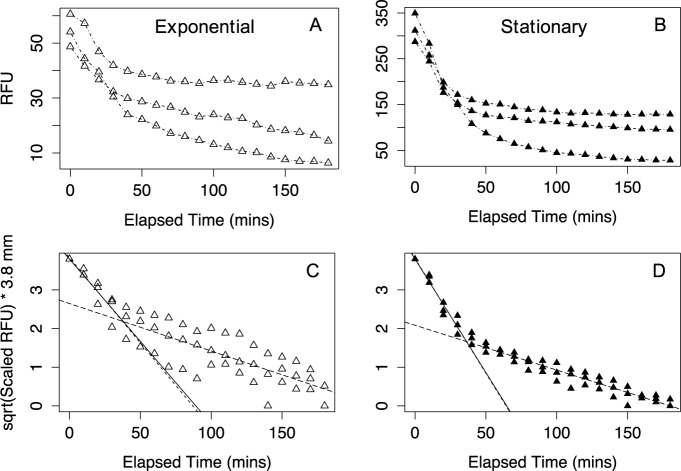
Raw data sinking assays and scaled sinking assays of *Skeletonema marinoi* RO5A triplicates in exponential and stationary phase. **(A)** Data points from a triplicated sinking assay *S*. *marinoi* in exponential phase plotted RFU *versus* Elapsed Time (minutes). **(B)** Triplicated sinking assay of *S*. *marinoi* in stationary phase plotted RFU *versus* Elapsed Time (minutes). **(C)** Exponential growth phase triplicates with sqrt(Scaled RFU) x 3.8 *versus* Elapsed Time (minutes). Data was fit in a segmented linear regression after breakpoint analysis. The long dashed line was the fitted linear regression of the first phase prior to the breakpoint, with no fixed intercept, while the nearly overlapping solid line is a linear regression prior to the breakpoint but using a fixed y-intercept of 3.8. The dashed line represents a linear regression of the second phase after the break point. The breakpoint is represented by the intersection of the dashed lines. **(D)** Stationary growth phase triplicates with sqrt(Scaled RFU) x 3.8 *versus* Elapsed Time (minutes). Data was fit in a segmented linear regression after breakpoint analysis. The long dashed line was the fitted linear regression of the first phase, with no fixed intercept, while the solid line is a fitted linear regression prior to the breakpoint but using a fixed y-intercept of 3.8. The dashed line represents a linear regression of the second phase following the breakpoint. The breakpoint is represented by the intersection of the dashed lines.

Results produced during the sinking trials of *S*. *marinoi* (Fig 2) suggested that cultures in exponential phase were more variable in the pattern of sinking rate compared to stationary phase. This result could be attributed to the lower cell suspension density of exponential cultures giving lower absolute signals more prone to experimental variation.

### Sinking rate analysis of unicellular diatoms

#### Sinking rate analysis of *Thalassiosira pseudonana* at exponential and stationary growth phase

**Fig 3:**
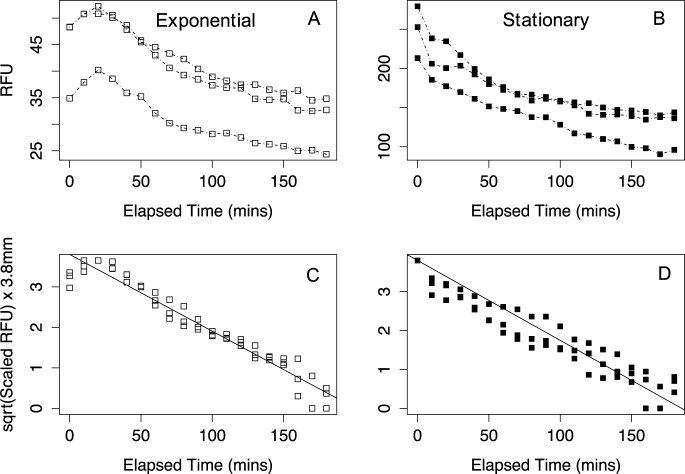
Raw data sinking assays and scaled sinking assays of *Thalassiosira pseudonana* CCMP1335 in exponential and stationary phase. **(A)** Data points from a triplicated sinking assay of *T*. *pseudonana* in exponential phase plotted as RFU *versus* Elapsed Time (minutes). **(B)** Triplicated sinking assay of *T*. *pseudonana* in stationary growth phase plotted as RFU *versus* Elapsed Time (minutes). **(C)** Exponential growth phase triplicates with sqrt(Scaled RFU) x 3.8 mm, *versus* Elapsed Time (minutes) fit with a single linear regression containing a fixed y-intercept of 3.8 mm. **(D)** Triplicated sinking assay of *T*. *pseudonana* in stationary growth phase plotted as sqrt(Scaled RFU) x 3.8 *versus* Elapsed Time (minutes) fit with a single linear regression containing a fixed y-intercept of 3.8.

The sinking assays of *T*. *pseudonana* in exponential phase and stationary phase (Fig 3) showed variation in absolute signal depending upon the initial density of the cell suspension. *T*. *pseudonana* in exponential phase (Fig 3A) showed an initial increase of measured fluorescence during the first thirty minutes of the sinking assay attributable to the slow relaxation of a phase of the organism's non-photochemical quenching mechanism [26]. We corrected for this as outlined in the materials and methods.

#### Sinking rate analysis of *Coscinodiscus radiatus* at exponential and stationary growth phase

**Fig 4:**
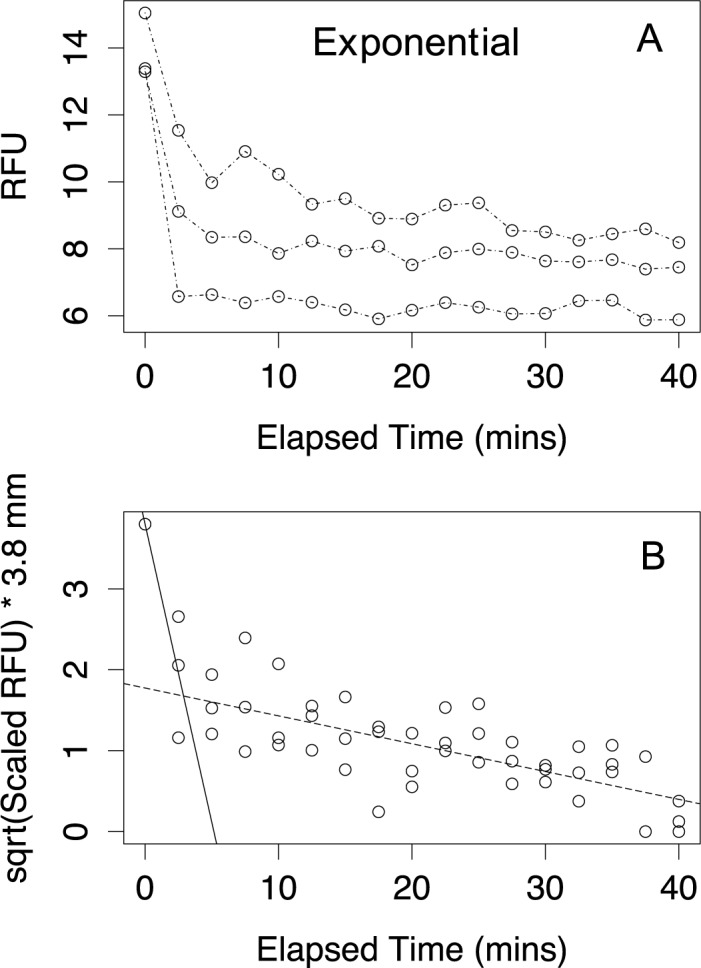
Raw data sinking assays and scaled sinking assays of *Coscinodiscus radiatus* CCMP312 during exponential phase in a 24 well-plate. **(A)** Data points from a triplicated sinking assay of *Coscinodiscus radiatus* in exponential phase plotted as RFU *versus* Elapsed Time (minutes). **(B)** Exponential growth phase triplicates with sqrt(Scaled RFU) x 3.8 *versus* Elapsed Time (minutes). Data was fit with a segmented linear regression after breakpoint analysis. The solid line is a linear regression of the first phase, prior to the break point, with a fixed y-intercept of 3.8. The dashed line represents a linear regression of the second phase after break point, while. The breakpoint is represented by the intersection of the lines.

Cultures of *C*. *radiatus* in exponential phase (Fig 4) required an alternate experimental timing as the large diatom sank the 3.8 mm depth of the well within ten minutes (data not presented). Sinking assays were therefore re-performed over a 40-minute time span with reads every 2.5 minutes. The sinking assay was also performed when cultures reached stationary phase but the segmented regression analysis found no breakpoint indicating that cells had already sunk prior to the second fluorescent reading at 2.5 minutes. We attempted to perform yet higher time resolutions by measuring fluorescence every 1 minute during a sinking assay but the data collected was scattered due to the spectrofluorometer’s technical limitations with relatively low fluorescence signals from low density cultures of *C*. *radiatus*.

Sinking rates of *C*. *radiatus* in exponential phase were significantly different when comparing measures in 24 vs. 96 well plates (S1 Statistics) (p value = 0.039), although well plate size did not have a detectable influence on sinking rates of the smaller *T*. *pseudonana*. We speculate that this is due to the large cell size of the *C*. *radiatus*. A 24 well-plate was used for the presented sinking rate determinations from *C*. *radiatus*. We caution researchers to take into consideration both time resolution and plate type during experimental design for accurate data collection.

### Effect of cell or filament length and phase of growth on sinking rate

Two-way fixed factor analysis of variance was performed on the first and second phase (if any) sinking rates (Table 1) (extracted from segmented regression preformed on each well in triplicate (S1 Statistics). After a logarithm transformation of first phase sinking rate, it was determined there was no interaction between the two independent factors in both ANOVAs (Growth Phase, Species). There was a significant difference between Species (p-value = 5.5x10^-8^) and Phase (p-value = 1.4 x 10^−4^).

**Table 1:** First and second (if any) phase sinking rates (m d^-1^) and associated amplitudes at exponential and stationary phases of growth.

Species	Filament/Cell Length(*μm*) ± *SE*		First Phase Sinking Rate (m d^-1^) ± *SE*		Second Phase Sinking Rate(m d^-1^) ± *SE*		First PhaseAmplitude ± *SE*		Second PhaseAmplitude ± *SE*	
	Expo	Stat	Expo	Stat	Expo	Stat	Expo	Stat	Expo	Stat
*T*. *pseudonana*	3.6 ± 0.23	5.876 ± 3.05	0.027 ± 0.001	0.030 ± 0.0004	N/A	N/A	1	1	N/A	N/A
*C*. *radiatus*	64.4 ± 4.2	N/A	1.06 * ± 0.532	N/A	0.014 ± 0.002	N/A	0.533 ± 0.965	N/A	0.467 ± 0.035	N/A
*S*. *marinoi*	19.3 ± 7.1	21.95 ± 3.61	0.061 ± 0.005	0.085±0.0035	0.018 ± 0.002	0.017 ± 0.0007	0.303 ± 0.046	0.451± 0.016	0.697 ± 0.046	0.548 ± 0.016

*Sinking rates calculated in sinking assay using 24 well-plate, with readings every 2.5 minutes for 40 minutes.

**Fig 5:**
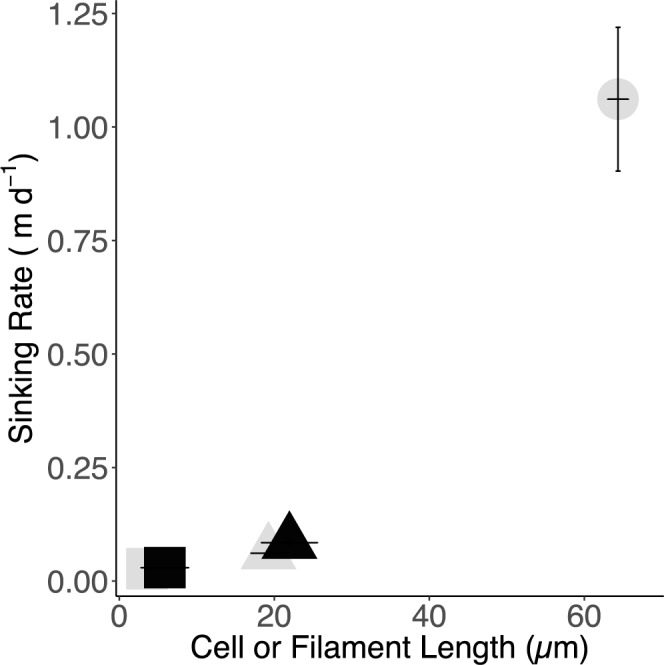
Effect of cell or filament length and growth phase on sinking rate. Graph depicts the relationship of sinking rate (m day^-1^) (Table 1) derived from the first phase of sinking (Figs 2,3 and 4) *versus* Cell or Filament Length (μm) with associated standard error bars. Three diatoms were studied; *Thalassiosira pseudonana* (square; *Skeletonema marinoi* (triangle) and *Coscinodiscus radiatus* (circle),. Sinking rates at different growth phases of each species were also studied, outlined grey shapes depict exponential phase while black filled shapes depict stationary phase.

We observed that diatom sinking rates, derived from the first phase of sinking, increased with cell size, and increased within a taxon from exponential to stationary phase (Fig 5).

We compared our experimental first phase sinking rates to literature values of sinking rates (Table 1). Comparison of diatom sinking rates after different growth light regimes using the SETCOL methodology [15] showed that *T*. *pseudonana* sank at 0.06 m d^-1^. This value falls within our range of experimental results. No literature values for sinking rates for *S*. *marinoi* were found. The sinking rates for growing cultures of the similar species *S*. *costatum* determined using the SETCOL method [16] were 0.33 m d^-1^, faster than the sinking rate experimentally determined during our sinking assay for *S*. *marinoi*. This difference could be accounted for by differences in species, filament length or experimental conditions in the *S*. *costatum* study. *Coscinodiscus* spp. showed a sinking rate of 1.90 ± 0.76 meters day^-1^ through SETCOL, within the range produced by our methods.

There are many factors that influence phytoplankton sinking rates including seawater density, cell size, temperature or metabolic status [35,36]. These factors can differ from study to study and variables must be taken into consideration during the attempt to compare to literature values. Overall, the validation experiments performed with the sinking assay accord with literature findings on the influences of growth phase and cell size on diatom sinking rate. In two of our three species we found a second, slower, phase of sinking that likely resulted from heterogeneity in the size or other properties of the cells in the cultures. We did not find literature evidence for such bi-modal sinking rates in other studies, but suggest that the relative amplitudes of the first and second phases likely reflects the relative abundances of sub-populations of the cells with different sinking rates.

The potential of this technique to be used as a sinking assay depends on the reproducibility and accuracy of replication. The success of this assay relies on the complete re-suspension of cells within culture placed in well. Failure to produce a completely homogenized culture within the well will lead to inconsistencies in the measurements of sinking rates as the average distance a cell sinks will vary. Power analysis [37] shows that the differences in sinking rates across the tested taxa and growth phases (Fig 5) were detected with very high confidence (~1) at a triplicate replication.

In these experiments, the well-plate was held in the body of spectrophotometer throughout the repeated readings of fluorescence during the sinking assay. This means that during the extent of the sinking assay temperature was not set to incubator temperature although in our case the incubator and room temperatures were similar. If the phytoplankton growth conditions were far from room temperature, temperature control during the sinking assay could be important. Furthermore, between the intervals of fluorescent reading, the well plate was held in the dark which could have influenced sinking rates of diatoms [14].

Throughout the sinking assay protocol, the cells in the well plate are exposed to multiple light regimes for varying periods of time during the movement of well plate in and out of laboratory. Since this assay relies on cellular fluorescence as a proxy for cell concentration, it will be sensitive to variables that influence culture fluorescence including light exposure history. Attempts were made to keep the timing and introduction of light consistent throughout experimental protocol, by using set time intervals for incubation and mixing. Phytoplankton could display species-specific responses to light history that would effect the accuracy of sinking rate calculations, as in the relaxation of sustained NPQ that we observed in our *T*. *pseudonana* experiments.

## Conclusions

Sinking rate is likely second only to growth rate as a key parameter determining the ecophysiology, trophic interactions and elemental cycling of phytoplankters. We present a simple approach to determine phytoplankton sinking rates across multiple culture samples in parallel using a plate spectrofluorometer. This sinking assay is inexpensive in time, energy and equipment compared to other methods for the analysis of sinking rates including SETCOL, side-reading fluorimeters and video surveillance. The plate based sinking assay nonetheless gave sinking rate estimates comparable to literature values from other approaches, with similar patterns of sinking rates vs. cell size and growth phase. We found limitations in the minimum culture density necessary for the sinking assay as noise from low fluorescence dilute suspensions obscures the sinking signal. As well, interactions with non-photochemical quenching mechanisms in a diatom, or in other phytoplankters, could mask the fluorescence signal resulting from sinking. The time resolution of measurements must be taken into consideration for optimal experimental design, particular for large or colonial taxa that sink rapidly. This time resolved fluorescence screen can be used to analyze phenotypic plasticity with growth phase, the responses of phytoplankton sinking rates to environmental variables, or for simple but informative phenotypic screens of mutant phytoplankton strains. We provide an annotated script, SinkWORX to facilitate the data processing (https://www.dropbox.com/sh/w03c2nt97rjk990/AACyg_nJrl75ztT9s6A89zT-a?dl=0).

## Supporting information

S1 Fig Representative micrographs. **(A)** *Thalassiosira pseudonana*. **(B)** *Coscinodiscus radiatus*. **(C)** *Skeletonema marinoi* filament of 6 cells.
(EPS)

S1 Script SinkWORX data import and analysis script. (ZIP)

S1 Statistics Data and statistical analyses scripts and data for well-plate effect and Proof of concept experiments. (ZIP)

S1 Data Fig 2A.RData. (ZIP)

S2 Data Fig 2B.RData. (ZIP)

S3 Data Fig 2C.RData. (ZIP)

S4 Data Fig 2D.RData. (ZIP)

S5 Data Fig 3A.RData. (ZIP)

S6 Data Fig 3B.RData. (ZIP)

S7 Data Fig 3C.RData. (ZIP)

S8 Data Fig 3D.RData. (ZIP)

S9 Data Fig 4A.RData. (ZIP)

S10 Data Fig 4B.RData. (ZIP)

S11 Data Fig 5.RData. (ZIP)
