# Correction: Sinking towards destiny: High throughput measurement of phytoplankton sinking rates through time-resolved fluorescence plate spectroscopy

**DOI:** 10.1371/journal.pone.0196624

**Published:** 2018-04-24

**Authors:** Catherine C. Bannon, Douglas A. Campbell

An incorrect version of the Supporting Information file S1 Script appears with the paper. Please see the correct version here.

## Supporting information

S1 Script SinkWORX data import and analysis script. (ZIP)
