# Supplementary material for: Sinking towards destiny: High throughput measurement of phytoplankton sinking rates through time-resolved fluorescence plate spectroscopy
Source: PLoS One. 2017 Oct 3;12(10):e0185166. doi: 10.1371/journal.pone.0185166 (PMC5626032; doi:10.1371/journal.pone.0185166)

S1.Stats.R

dcampbel

Thu Sep 14 12:37:33 2017

```r
#Statistical analyses of Well Effects for Bannon & Campbell, PLoSOne 2017

#install a package "here" to manage file access paths
library(here)
```

```
## here() starts at /Users/dcampbel/Dropbox/Skeletonema Honours 16-17/Manuscript/Statistics
```

```r
library(car) # calls the correct libraries
library(psych)
```

```
##
## Attaching package: 'psych'

## The following object is masked from 'package:car':
##
##     logit
```

```r
library(sciplot)

#################Open WellEffect_Stats.csv within Statistic folder

WellEffect_Stats <- read.file.csv(here("WellEffect_Stats.csv"))

names(WellEffect_Stats)
```

```
##  [1] "Species"            "Phase"              "Plate"
##  [4] "SecondPhaseRate"    "SecondPhaseRate.SE" "FirstPhaseRate"
##  [7] "FirstPhaseRate.SE"  "FirstAmplitude"     "FirstAmplitude.SE"
## [10] "SecondAmplitude"    "SecondAmplitude.SE" "Notes"
```

```r
#describeBy(Slope, list (Phase, Species, Wellplate)) # descriptive stats
FirstSinking <- as.numeric(WellEffect_Stats$FirstPhaseRate)
Plate <- as.factor(WellEffect_Stats$Plate)
Species <- as.factor(WellEffect_Stats$Species)
Phase <- as.factor(WellEffect_Stats$Phase)

#par(mar = c(7, 4, 2, 2) + 3)
#boxplot(FirstSinking~Plate*Phase*Species, ylab="Sinking Rate m/day", las=3) # boxplot

#subsetting species
Coscis <- subset(WellEffect_Stats,Species =="Coscis") # splits the file to analyze separately for each
Pseudo <- subset(WellEffect_Stats,Species =="Pseudo")

#subsetting stationary and exponential phase in species
Pseudo.Expo <- subset(Pseudo,Phase =="Expo")
Pseudo.Stat <- subset(Pseudo,Phase =="Stat")

#subsetting stationary and exponential phase in coscis
Coscis.Expo <- subset(Coscis,Phase =="Expo")
Coscis.Stat <- subset(Coscis,Phase =="Stat")
```

```r
par(new = TRUE)
```

```
## Warning in par(new = TRUE): calling par(new=TRUE) with no plot
```

```r
par(mfrow=c(2,2))
describeBy(FirstSinking, Plate) # lets us look at descriptive stats
```

```
##
##  Descriptive statistics by group
## group: ninetysix
##    vars  n mean   sd median trimmed  mad  min  max range skew kurtosis
## X1    1 12 0.16 0.17   0.07    0.14 0.08 0.02 0.51  0.49 0.74    -1.03
##      se
## X1 0.05
## --------------------------------------------------------
## group: twentyfour
##    vars  n mean   sd median trimmed  mad  min  max range skew kurtosis
## X1    1 12 0.43 0.51   0.21    0.36 0.28 0.02 1.52   1.5 0.81    -0.77
##      se
## X1 0.15
```

```r
######################################################################
##########################Second Phase################################
######################################################################
#plate effect in coscis expo
coscis.expo <- aov(SecondPhaseRate~Plate, data = Coscis.Expo ) # model for 2-way anova, interaction inc
summary(coscis.expo)
```

```
##             Df  Sum Sq Mean Sq F value Pr(>F)
## Plate        1 0.01349 0.01349   0.456  0.537
## Residuals    4 0.11841 0.02960
```

```r
coscis.expo.resid=resid(coscis.expo) # generating residuals, test normality on resisdualsSecondPhaseRat
shapiro.test(coscis.expo.resid) #normal
```

```
##
##  Shapiro-Wilk normality test
##
## data:  coscis.expo.resid
## W = 0.86724, p-value = 0.2154
```

```r
boxplot(SecondPhaseRate~Plate, xlab="Plate", ylab="Second Sinking Rate", main="Coscis Exponential", data

#plate effect in coscis stat
coscis.stat <- aov(SecondPhaseRate~Plate, data = Coscis.Stat ) # model for 2-way anova, interaction inc
summary(coscis.stat)
```

```
##             Df   Sum Sq  Mean Sq F value Pr(>F)
## Plate        1 0.003334 0.003334   2.119  0.219
## Residuals    4 0.006294 0.001574
```

```r
coscis.stat.resid=resid(coscis.stat) # generating residuals, test normality on resisduals
shapiro.test(coscis.stat.resid)
```

```
##
##  Shapiro-Wilk normality test
##
```

```
## data:  coscis.stat.resid
## W = 0.88002, p-value = 0.2691
```

```
boxplot(SecondPhaseRate~Plate, xlab="Plate", ylab="Second Sinking Rate", main="Coscis Stationary", data



#######################################################################
############################First Phase ###############################
#######################################################################
#plate effect in pseudo expo
pseudo.expo <- aov(FirstPhaseRate~Plate, data = Pseudo.Expo ) # model for 1-way anova
summary(pseudo.expo)
```

```
##             Df    Sum Sq  Mean Sq F value Pr(>F)
## Plate        1 8.090e-05 8.09e-05   7.099 0.0561 .
## Residuals    4 4.558e-05 1.14e-05
## ---
## Signif. codes:  0 '***' 0.001 '**' 0.01 '*' 0.05 '.' 0.1 ' ' 1
```

```
pseudo.expo.resid=resid(pseudo.expo) # generating residuals, test normality on resisduals
shapiro.test(pseudo.expo.resid) # normality test, normal
```

```
##
##  Shapiro-Wilk normality test
##
## data:  pseudo.expo.resid
## W = 0.89507, p-value = 0.3456
```

```
boxplot(FirstPhaseRate~Plate, xlab="Plate", ylab="First Sinking Rate", main="Pseudo Exponential", data =

#plate effect in pseudo stat
pseudo.stat <- aov(FirstPhaseRate~Plate, data = Pseudo.Stat ) # model for 1-way anova
pseudo.stat.resid=resid(pseudo.stat) # generating residuals, test normality on resisduals
shapiro.test(pseudo.stat.resid) #normal
```

```
##
##  Shapiro-Wilk normality test
##
## data:  pseudo.stat.resid
## W = 0.87705, p-value = 0.2558
```

```
boxplot(FirstPhaseRate~Plate, xlab="Plate", ylab="First Sinking Rate", main="Pseudo Stationary", data =
```

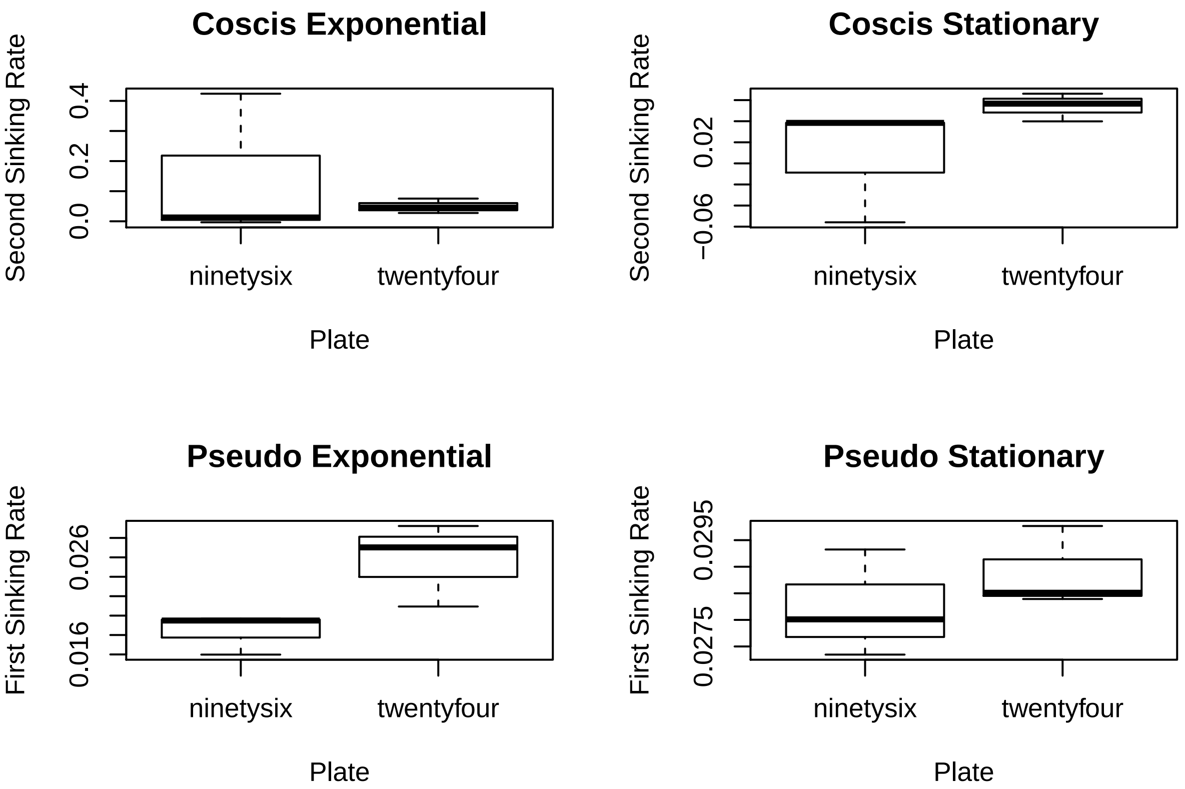


```
#plate effect in coscis expo
coscis.expo <- aov(FirstPhaseRate~Plate, data = Coscis.Expo ) # model for 1-way anova
summary(coscis.expo)
```

```
##             Df Sum Sq Mean Sq F value Pr(>F)
## Plate        1 0.9413  0.9413   9.142  0.039 *
## Residuals    4 0.4118  0.1030
## ---
## Signif. codes:  0 '***' 0.001 '**' 0.01 '*' 0.05 '.' 0.1 ' ' 1
```

```
coscis.expo.resid=resid(coscis.expo) # generating residuals, test normality on resisduals
shapiro.test(coscis.expo.resid) #normal
```

```
##
##  Shapiro-Wilk normality test
##
## data:  coscis.expo.resid
## W = 0.97387, p-value = 0.9174
```

```
boxplot(FirstPhaseRate~Plate, xlab="Plate", ylab="First Sinking Rate", main="Coscis Exponential", data

#plate effect in coscis stat
coscis.stat <- aov(FirstPhaseRate~Plate, data = Coscis.Stat )# model for 1-way anova
summary(coscis.stat)
```

```
##             Df Sum Sq Mean Sq F value Pr(>F)
## Plate        1 0.1224 0.12236   1.841  0.246
## Residuals    4 0.2658 0.06645
```

```
coscis.stat.resid=resid(coscis.stat) # generating residuals, test normality on resisduals
shapiro.test(coscis.stat.resid)
```

```
##
##  Shapiro-Wilk normality test
##
## data:  coscis.stat.resid
## W = 0.85451, p-value = 0.1711
```

```r
boxplot(FirstPhaseRate~Plate, xlab="Plate", ylab="First Sinking Rate", main="Coscis Stationary", data =
```

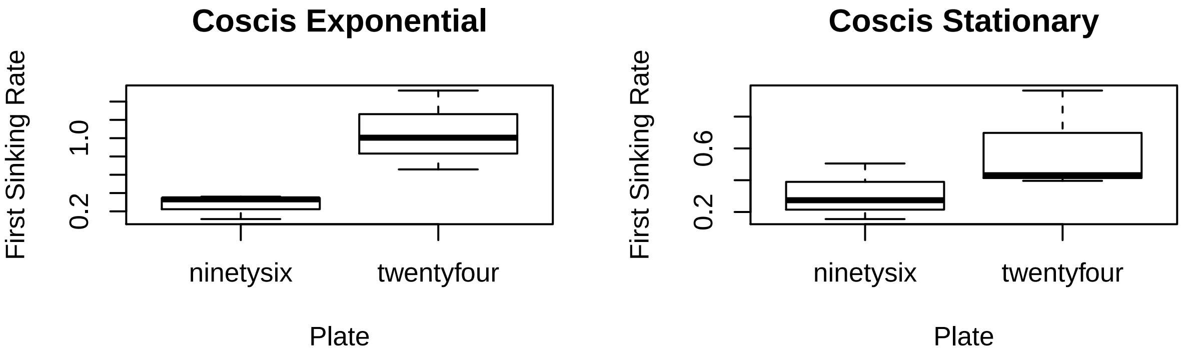

Supplement: S1 Statistics — (ZIP) [file pone.0185166.s003.zip › Statistics/S1.Stats.pdf]
