# Supplementary material for: Sinking towards destiny: High throughput measurement of phytoplankton sinking rates through time-resolved fluorescence plate spectroscopy
Source: PLoS One. 2017 Oct 3;12(10):e0185166. doi: 10.1371/journal.pone.0185166 (PMC5626032; doi:10.1371/journal.pone.0185166)
Supplement: S1 Statistics — (ZIP) [file pone.0185166.s003.zip › Statistics/S2.Stats.pdf]

S2.Stats.R

dcampbel

Thu Sep 14 12:44:17 2017

```
#Statistical analyses of Proof of Concept Experiments for Bannon & Campbell, PLoSOne 2017
#made by Catherine Bannon & Douglas Campbell

#install a package "here" to manage file access paths
library(here)
```

```
## here() starts at /Users/dcampbel/Dropbox/Skeletonema Honours 16-17/Manuscript/Statistics
```

```
library(car) # for leveneTest
library(psych) # for describeBy
```

```
##
## Attaching package: 'psych'

## The following object is masked from 'package:car':
##
##     logit
```

```
library(sciplot) # lineplot.CI function, visualize interactio
library(pwr)

#################Retrieve Manuscript Proof of Concept Experiments CSV
#################Open Manuscript_Stats.csv within Statistic folder
Manuscript_Stats <- read.file.csv(here("Manuscript_Stats.csv"))
attach(Manuscript_Stats)
names(Manuscript_Stats)
```

```
##  [1] "Species"             "Phase"               "SecondPhaseRate"
##  [4] "SecondPhaseRate.SE" "FirstPhaseRate"      "FirstPhaseRate.SE"
##  [7] "FirstAmplitude"     "FirstAmplitude.SE"   "SecondAmplitude"
## [10] "SecondAmplitude.SE" "Notes"
```

```
###########FirstPhaseRate Comparison
describeBy(FirstPhaseRate, list (Phase, Species))
```

```
##
##  Descriptive statistics by group
## : Expo
## : Coscis
##    vars n mean   sd median trimmed  mad  min  max range skew kurtosis   se
## X1    1 3 1.06 0.43   1.01    1.06 0.51 0.66 1.52  0.86 0.13    -2.33 0.25
## --------------------------------------------------------
## : Stat
## : Coscis
## NULL
## --------------------------------------------------------
## : Expo
## : Pseudo
##    vars n mean sd median trimmed mad  min  max range skew kurtosis se
## X1    1 3 0.03  0   0.03    0.03   0 0.03 0.03     0 0.38    -2.33  0
```

```
## --------------------------------------------------------
## : Stat
## : Pseudo
##    vars n mean sd median trimmed mad  min  max range  skew kurtosis se
## X1    1 3 0.03  0   0.03    0.03   0 0.03 0.03  0.01 -0.26    -2.33  0
## --------------------------------------------------------
## : Expo
## : Skele
##    vars n mean   sd median trimmed mad  min  max range skew kurtosis   se
## X1    1 3 0.06 0.02   0.05    0.06   0 0.05 0.08  0.03 0.38    -2.33 0.01
## --------------------------------------------------------
## : Stat
## : Skele
##    vars n mean   sd median trimmed  mad  min max range  skew kurtosis   se
## X1    1 3 0.08 0.01   0.08    0.08 0.02 0.07 0.1  0.02 -0.01    -2.33 0.01
```

```
boxplot(FirstPhaseRate~Phase*Species, xlab="Phase x Species", ylab="Sinking Rate") # boxplot
```

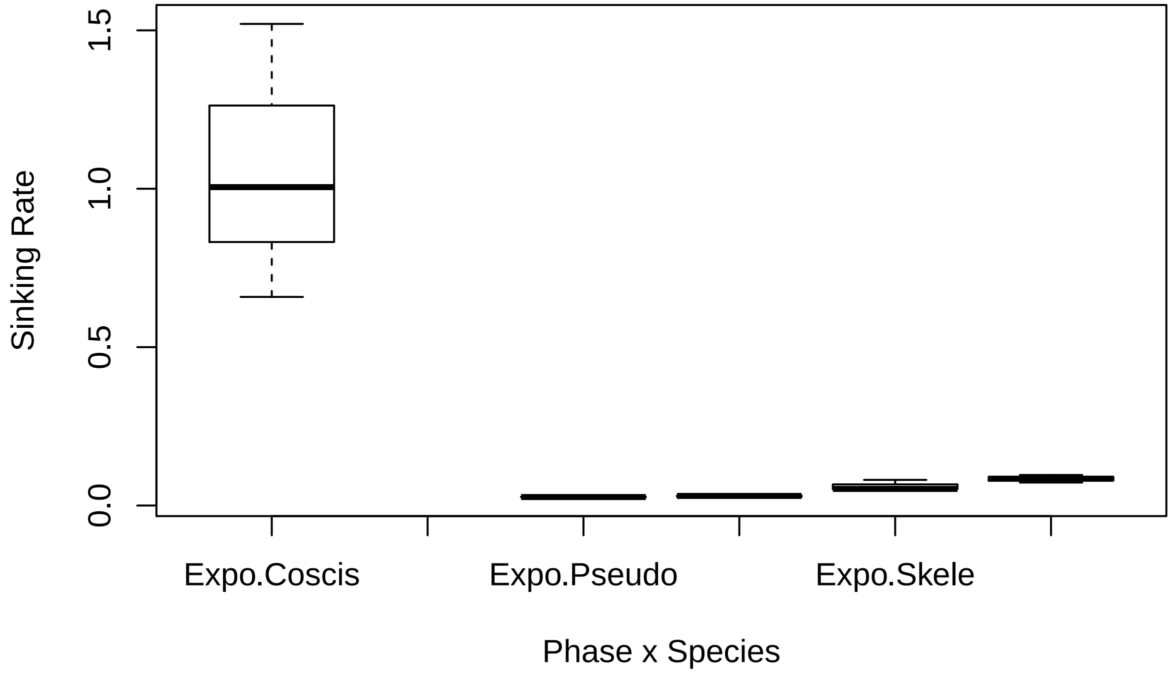


```
lineplot.CI(x.factor=Phase, response=FirstPhaseRate, group=Species, trace.label="Species", xlab="Phase"
```

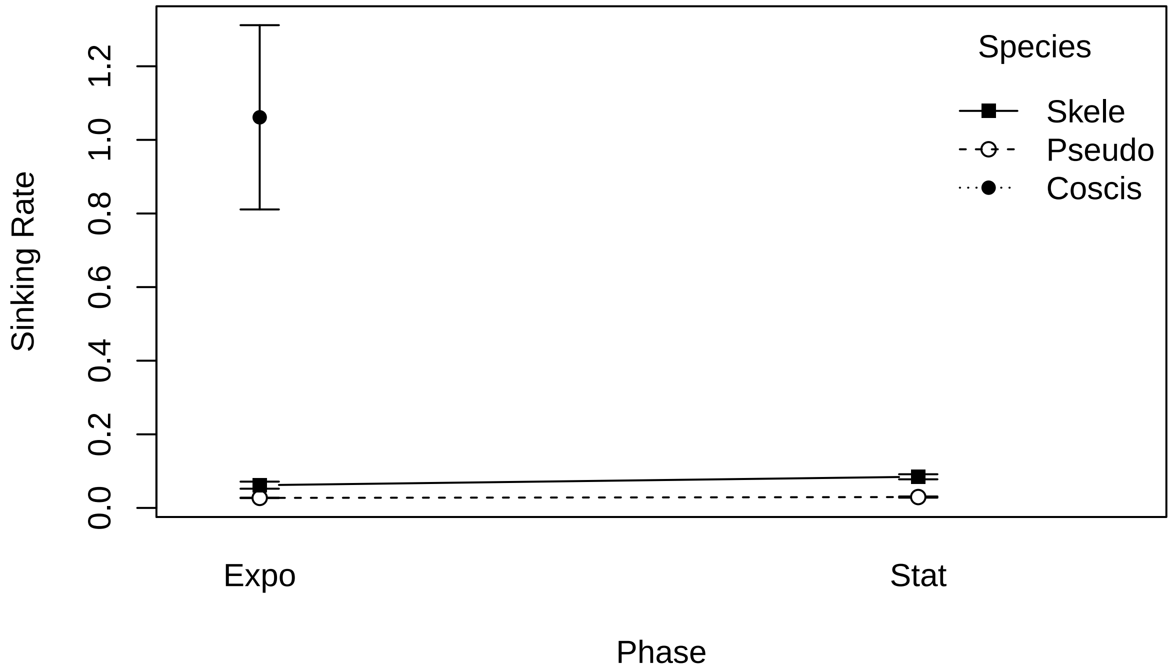


```
leveneTest (FirstPhaseRate, interaction(Phase, Species)) #HoV test
```

```
## Levene's Test for Homogeneity of Variance (center = median)
##       Df F value Pr(>F)
## group  4  3.4516  0.051 .
##       10
## ---
## Signif. codes:  0 '***' 0.001 '**' 0.01 '*' 0.05 '.' 0.1 ' ' 1
```

```
Firstsink.fit <- aov(FirstPhaseRate~Phase*Species) # model for 2-way anova, interaction included
summary(Firstsink.fit) #Phase effect, Species effect and interaction
```

```
##               Df Sum Sq Mean Sq F value  Pr(>F)
## Phase          1 0.3835  0.3835  10.176 0.00965 **
## Species        2 2.0736  1.0368  27.511 8.6e-05 ***
## Phase:Species  1 0.0003  0.0003   0.008 0.92883
## Residuals     10 0.3769  0.0377
## ---
## Signif. codes:  0 '***' 0.001 '**' 0.01 '*' 0.05 '.' 0.1 ' ' 1
```

```
Firstsink.resid=resid(Firstsink.fit) # generating residuals, test normality on resisduals
qqnorm(Firstsink.resid) # plotting residuals for visual test of normality
qqline(Firstsink.resid)
```

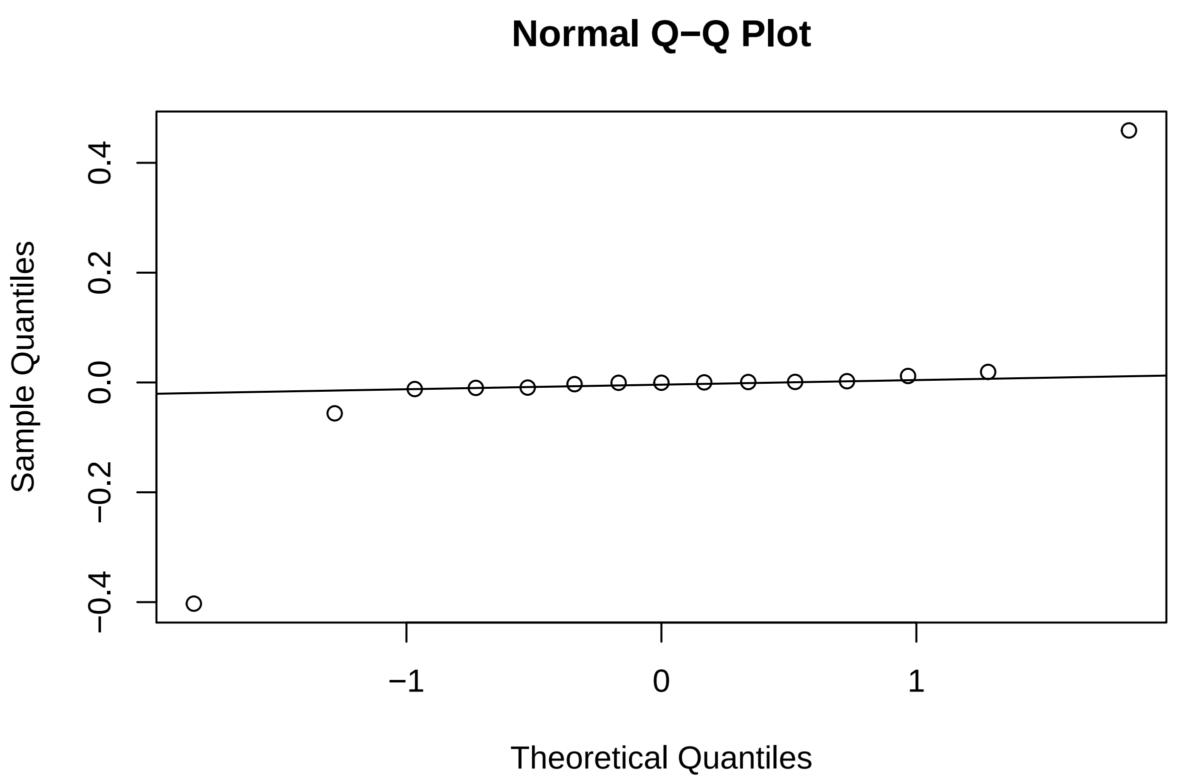


```r
shapiro.test(Firstsink.resid) # normality test, not normal
```

```
##
##  Shapiro-Wilk normality test
##
## data:  Firstsink.resid
## W = 0.60805, p-value = 3.022e-05
```

```r
TukeyHSD(Firstsink.fit)
```

```
##   Tukey multiple comparisons of means
##     95% family-wise confidence level
##
## Fit: aov(formula = FirstPhaseRate ~ Phase * Species)
##
## $Phase
##                  diff        lwr         upr    p adj
## Stat-Expo -0.3263844 -0.5543556 -0.09841319 0.009654
##
## $Species
##                     diff        lwr        upr     p adj
## Pseudo-Coscis -0.86968970 -1.2459859 -0.4933935 0.0002276
## Skele-Coscis  -0.82488322 -1.2011794 -0.4485870 0.0003479
## Skele-Pseudo   0.04480648 -0.2624381  0.3520510 0.9163926
##
## $`Phase:Species`
##                                  diff        lwr        upr     p adj
## Stat:Coscis-Expo:Coscis            NA         NA         NA        NA
## Expo:Pseudo-Expo:Coscis -1.03401423 -1.5845533 -0.4834751 0.0006876
## Stat:Pseudo-Expo:Coscis -1.03174958 -1.5822887 -0.4812105 0.0006999
```

```
## Expo:Skele-Expo:Coscis  -0.99947327 -1.5500124 -0.4489342 0.0009029
## Stat:Skele-Expo:Coscis  -0.97667757 -1.5272167 -0.4261385 0.0010840
## Expo:Pseudo-Stat:Coscis          NA         NA         NA        NA
## Stat:Pseudo-Stat:Coscis          NA         NA         NA        NA
## Expo:Skele-Stat:Coscis           NA         NA         NA        NA
## Stat:Skele-Stat:Coscis           NA         NA         NA        NA
## Stat:Pseudo-Expo:Pseudo  0.00226465 -0.5482744  0.5528037 1.0000000
## Expo:Skele-Expo:Pseudo   0.03454096 -0.5159981  0.5850800 0.9999077
## Stat:Skele-Expo:Pseudo   0.05733666 -0.4932024  0.6078757 0.9989136
## Expo:Skele-Stat:Pseudo   0.03227631 -0.5182628  0.5828154 0.9999339
## Stat:Skele-Stat:Pseudo   0.05507201 -0.4954671  0.6056111 0.9991046
## Stat:Skele-Expo:Skele    0.02279570 -0.5277434  0.5733348 0.9999882
```

```
###############LOG TRANSFORM DATA#####
Manuscript_Stats$log.FR <- log10(Manuscript_Stats$FirstPhaseRate)
boxplot(log.FR~Phase*Species, xlab="Phase x Species", ylab="Sinking Rate", data=Manuscript_Stats) # box
```

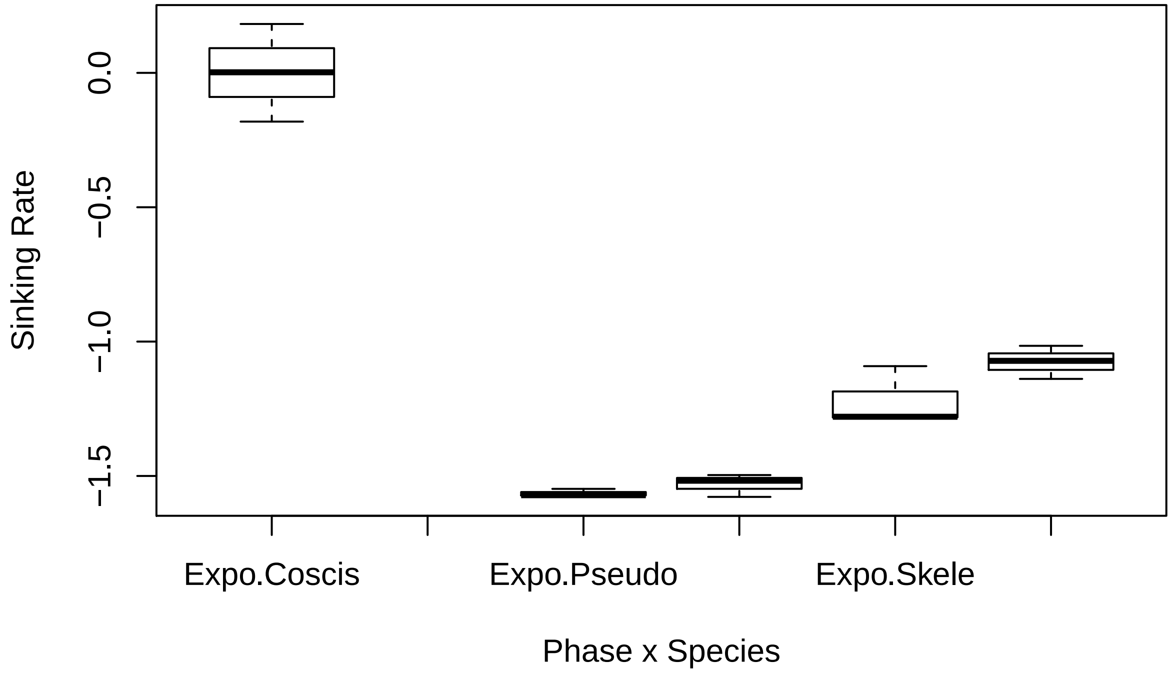


```
lineplot.CI(x.factor=Phase, response=log.FR, group=Species, trace.label="Species", xlab="Phase", ylab="
```

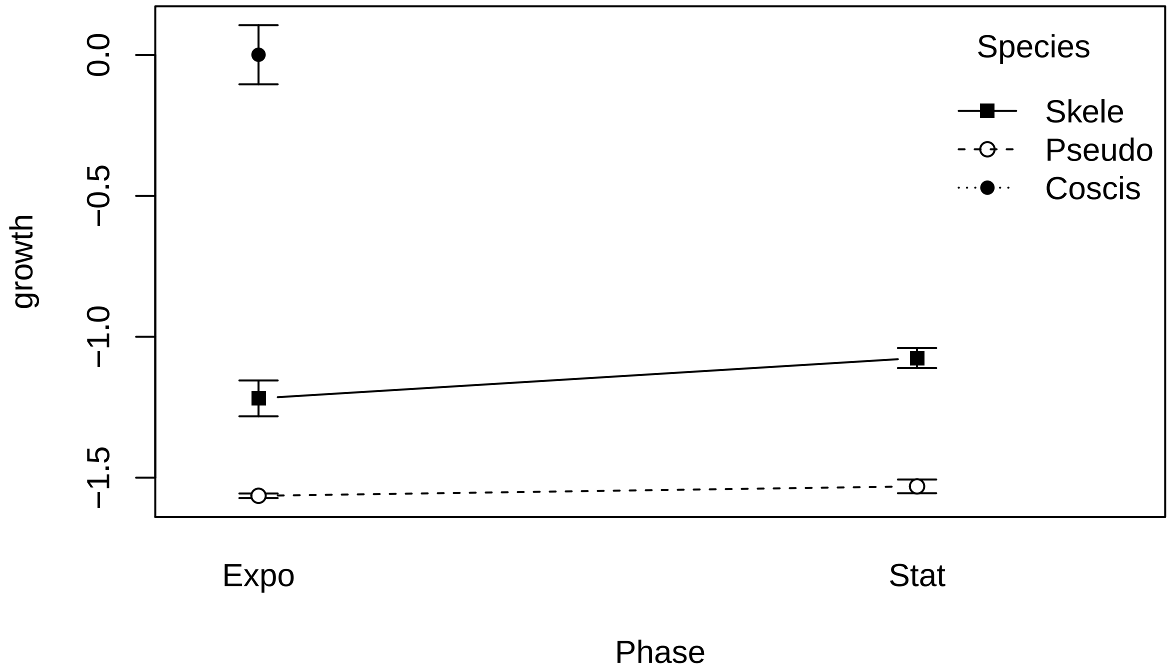


```
leveneTest (Manuscript_Stats$log.FR, interaction(Phase, Species)) #HoV test
```

```
## Levene's Test for Homogeneity of Variance (center = median)
##       Df F value Pr(>F)
## group  4  1.1397 0.3923
##       10
```

```
logsink.fit <- aov(log.FR~Phase*Species, data=Manuscript_Stats) # model for 2-way anova, interaction in
summary(logsink.fit) #Phase effect, Species effect and interaction
```

```
##               Df Sum Sq Mean Sq F value   Pr(>F)
## Phase          1  0.508  0.5085  49.948 3.43e-05 ***
## Species        2  4.358  2.1791 214.066 6.19e-09 ***
## Phase:Species  1  0.009  0.0090   0.886    0.369
## Residuals     10  0.102  0.0102
## ---
## Signif. codes:  0 '***' 0.001 '**' 0.01 '*' 0.05 '.' 0.1 ' ' 1
```

```
sink.resid=resid(logsink.fit) # generating residuals, test normality on resisduals
qqnorm(sink.resid) # plotting residuals for visual test of normality
qqline(sink.resid)
```

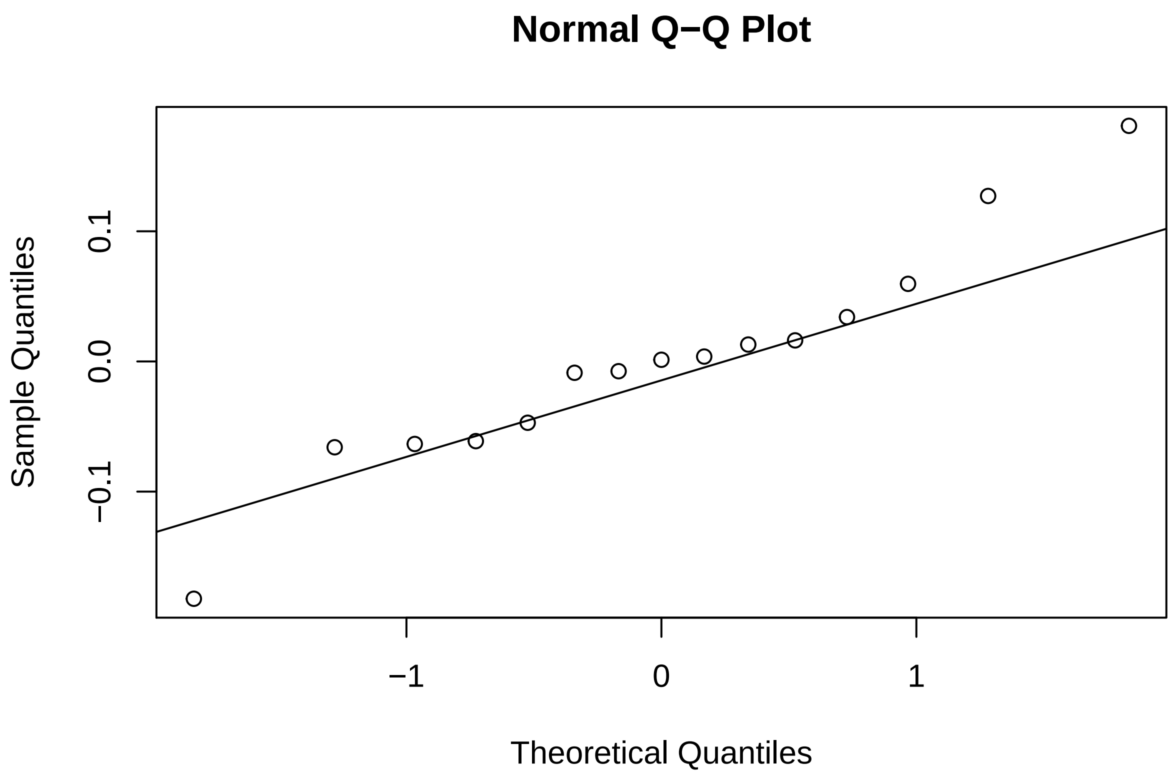


```
shapiro.test(sink.resid) # normality test, not normal
```

```
##
##  Shapiro-Wilk normality test
##
## data:  sink.resid
## W = 0.94713, p-value = 0.4804
```

```
####### post hoc testing##########
TukeyHSD(logsink.fit)
```

```
##   Tukey multiple comparisons of means
##     95% family-wise confidence level
##
## Fit: aov(formula = log.FR ~ Phase * Species, data = Manuscript_Stats)
##
## $Phase
##                 diff        lwr        upr    p adj
## Stat-Expo -0.375818 -0.4943021 -0.2573339 3.43e-05
##
## $Species
##                     diff        lwr        upr    p adj
## Pseudo-Coscis -1.3605198 -1.5560933 -1.1649464 0.00e+00
## Skele-Coscis  -0.9599754 -1.1555488 -0.7644019 3.00e-07
## Skele-Pseudo   0.4005445  0.2408594  0.5602295 1.16e-04
##
## $`Phase:Species`
##                                diff         lwr        upr     p adj
## Stat:Coscis-Expo:Coscis          NA          NA         NA        NA
## Expo:Pseudo-Expo:Coscis -1.5651482 -1.85128135 -1.2790150 0.0000000
```

```
## Stat:Pseudo-Expo:Coscis -1.5317095 -1.81784265 -1.2455763 0.0000001
## Expo:Skele-Expo:Coscis  -1.2194461 -1.50557928 -0.9333129 0.0000004
## Stat:Skele-Expo:Coscis  -1.0763226 -1.36245582 -0.7901895 0.0000014
## Expo:Pseudo-Stat:Coscis         NA          NA         NA        NA
## Stat:Pseudo-Stat:Coscis         NA          NA         NA        NA
## Expo:Skele-Stat:Coscis          NA          NA         NA        NA
## Stat:Skele-Stat:Coscis          NA          NA         NA        NA
## Stat:Pseudo-Expo:Pseudo  0.0334387 -0.25269449  0.3195719 0.9981194
## Expo:Skele-Expo:Pseudo   0.3457021  0.05956889  0.6318353 0.0167462
## Stat:Skele-Expo:Pseudo   0.4888255  0.20269235  0.7749587 0.0014558
## Expo:Skele-Stat:Pseudo   0.3122634  0.02613020  0.5983966 0.0308692
## Stat:Skele-Stat:Pseudo   0.4553868  0.16925365  0.7415200 0.0024986
## Stat:Skele-Expo:Skele    0.1431235 -0.14300973  0.4292566 0.5397603
```

```r
summary(logsink.fit)
```

```
##                Df Sum Sq Mean Sq F value   Pr(>F)
## Phase           1  0.508  0.5085  49.948 3.43e-05 ***
## Species         2  4.358  2.1791 214.066 6.19e-09 ***
## Phase:Species   1  0.009  0.0090   0.886    0.369
## Residuals      10  0.102  0.0102
## ---
## Signif. codes:  0 '***' 0.001 '**' 0.01 '*' 0.05 '.' 0.1 ' ' 1
```

```r
#power test for Phase
pwr.anova.test(k = 2, n = 3, f = 35.459, sig.level = 0.05)
```

```
##
##      Balanced one-way analysis of variance power calculation
##
##               k = 2
##               n = 3
##               f = 35.459
##       sig.level = 0.05
##           power = 1
##
## NOTE: n is number in each group
```

```r
#power test for Species
pwr.anova.test(k = 3, n = 6, f = 136.539, sig.level = 0.05)
```

```
##
##      Balanced one-way analysis of variance power calculation
##
##               k = 3
##               n = 6
##               f = 136.539
##       sig.level = 0.05
##           power = 1
##
## NOTE: n is number in each group
```

```r
###########SecondPhaseRate Comparison
describeBy(SecondPhaseRate, list (Phase, Species)) # descriptive stats
```

```
## Warning in min(x, na.rm = na.rm): no non-missing arguments to min;
## returning Inf
```

```
## Warning in max(x, na.rm = na.rm): no non-missing arguments to max;
## returning -Inf

## Warning in min(x, na.rm = na.rm): no non-missing arguments to min;
## returning Inf

## Warning in max(x, na.rm = na.rm): no non-missing arguments to max;
## returning -Inf

##
##  Descriptive statistics by group
## : Expo
## : Coscis
##    vars n mean   sd median trimmed  mad  min  max range skew kurtosis   se
## X1    1 3 0.05 0.02   0.05    0.05 0.03 0.03 0.08  0.05 0.16    -2.33 0.01
## --------------------------------------------------------
## : Stat
## : Coscis
## NULL
## --------------------------------------------------------
## : Expo
## : Pseudo
##    vars n mean sd median trimmed mad min  max range skew kurtosis se
## X1    1 0  NaN NA     NA     NaN  NA Inf -Inf  -Inf   NA       NA NA
## --------------------------------------------------------
## : Stat
## : Pseudo
##    vars n mean sd median trimmed mad min  max range skew kurtosis se
## X1    1 0  NaN NA     NA     NaN  NA Inf -Inf  -Inf   NA       NA NA
## --------------------------------------------------------
## : Expo
## : Skele
##    vars n mean   sd median trimmed mad  min  max range  skew kurtosis se
## X1    1 3 0.02 0.01   0.02    0.02   0 0.01 0.02  0.01 -0.35    -2.33  0
## --------------------------------------------------------
## : Stat
## : Skele
##    vars n mean sd median trimmed mad  min  max range  skew kurtosis se
## X1    1 3 0.02  0   0.02    0.02   0 0.02 0.02     0 -0.03    -2.33  0
```

```
boxplot(SecondPhaseRate~Phase*Species, xlab="Phase x Species", ylab="Sinking Rate") # boxplot
```

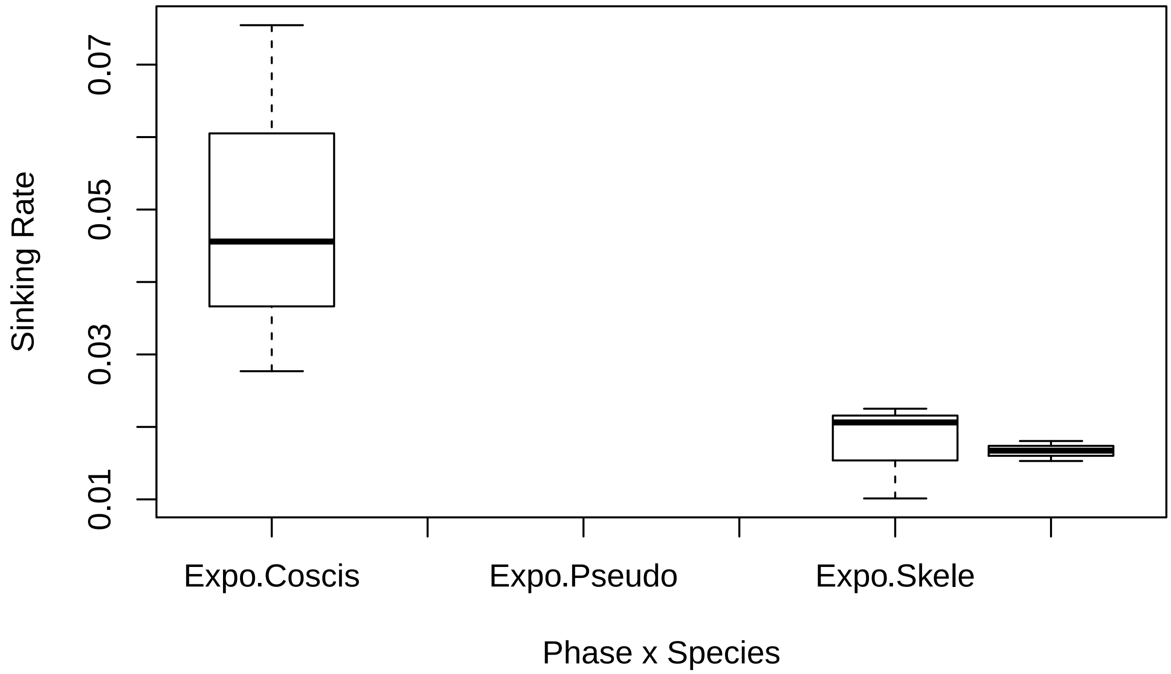


```
#lineplot.CI(x.factor=Phase, response=SecondPhaseRate, group=Species, trace.label="Species", xlab="Phas
leveneTest (SecondPhaseRate, interaction(Phase, Species)) #HoV test
```

```
## Levene's Test for Homogeneity of Variance (center = median)
##       Df F value Pr(>F)
## group  2  2.1786 0.1944
##        6
```

```
secondsink.fit <- aov(SecondPhaseRate~Phase*Species) # model for 2-way anova, interaction included
summary(secondsink.fit) #Phase effect, Species effect and interaction
```

```
##             Df    Sum Sq   Mean Sq F value Pr(>F)
## Phase        1 0.0005759 0.0005759   2.749 0.1484
## Species      1 0.0015185 0.0015185   7.249 0.0359 *
## Residuals    6 0.0012569 0.0002095
## ---
## Signif. codes:  0 '***' 0.001 '**' 0.01 '*' 0.05 '.' 0.1 ' ' 1
## 6 observations deleted due to missingness
```

```
sink.resid=resid(secondsink.fit) # generating residuals, test normality on resisduals
qqnorm(sink.resid) # plotting residuals for visual test of normality
qqline(sink.resid)
```

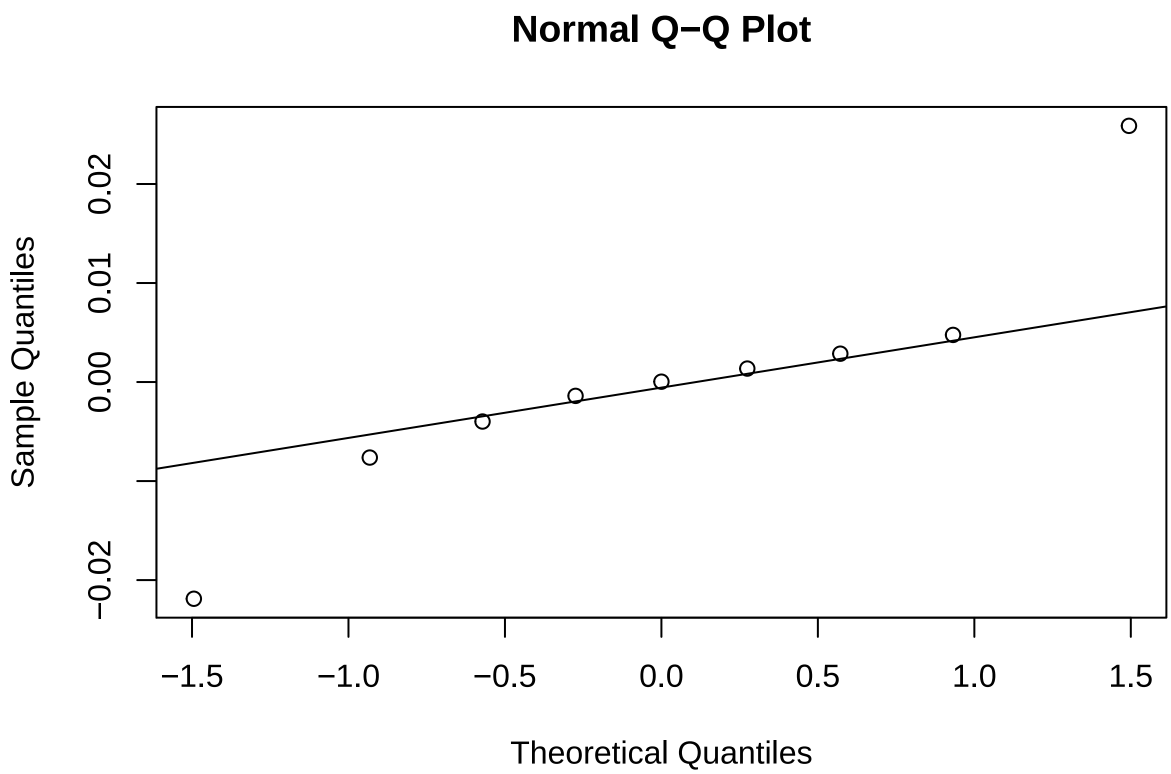


```r
shapiro.test(sink.resid) # normality test, not normal
```

```
##
##  Shapiro-Wilk normality test
##
## data:  sink.resid
## W = 0.90612, p-value = 0.2896
```

```r
TukeyHSD(secondsink.fit)
```

```
##   Tukey multiple comparisons of means
##     95% family-wise confidence level
##
## Fit: aov(formula = SecondPhaseRate ~ Phase * Species)
##
## $Phase
##                  diff         lwr         upr     p adj
## Stat-Expo -0.01696958 -0.04201187 0.008072696 0.1483671
##
## $Species
##                    diff         lwr         upr     p adj
## Skele-Coscis -0.02386263 -0.04890491 0.001179651 0.0585087
```
