# Supplementary material for: Correction: Sinking towards destiny: High throughput measurement of phytoplankton sinking rates through time-resolved fluorescence plate spectroscopy
Source: PLoS One. 2018 Apr 24;13(4):e0196624. doi: 10.1371/journal.pone.0196624 (PMC5915776; doi:10.1371/journal.pone.0196624)
Supplement: S1 Script — (ZIP) [file pone.0196624.s001.zip › SinkWORX/ManualMaterial/SinkWorx summary flowchart.pptx]

## Slide 1
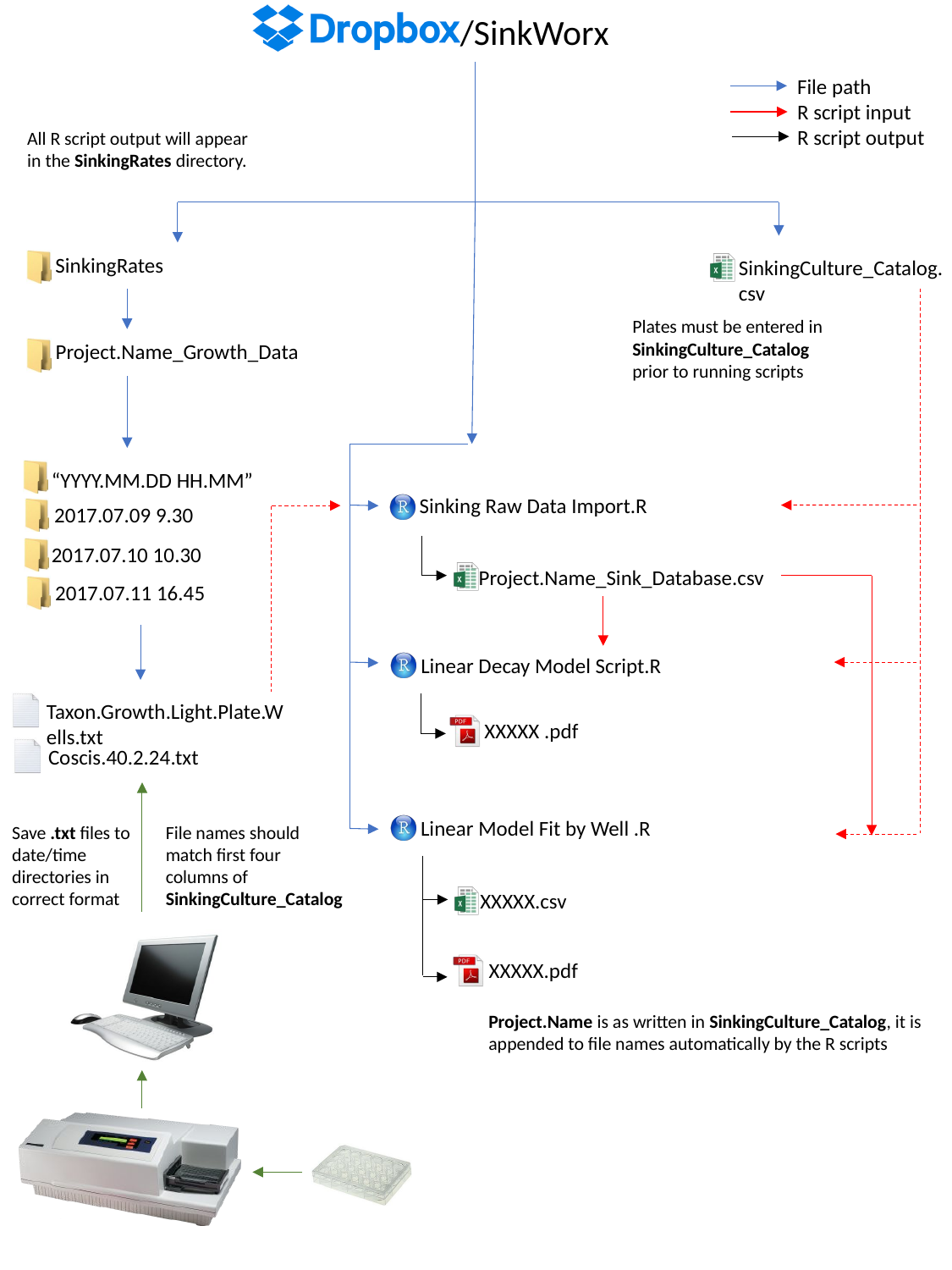

/SinkWorx
 File path
 R script input
 R script output
All R script output will appear in the SinkingRates directory.
SinkingRates
SinkingCulture_Catalog.csv
Plates must be entered in SinkingCulture_Catalog prior to running scripts
Project.Name_Growth_Data
“YYYY.MM.DD HH.MM”
Sinking Raw Data Import.R
2017.07.09 9.30
2017.07.10 10.30
Project.Name_Sink_Database.csv
2017.07.11 16.45
Linear Decay Model Script.R
Taxon.Growth.Light.Plate.Wells.txt
XXXXX .pdf
Coscis.40.2.24.txt
Linear Model Fit by Well .R
Save .txt files to date/time directories in correct format
File names should match first four columns of SinkingCulture_Catalog
XXXXX.csv
XXXXX.pdf
Project.Name is as written in SinkingCulture_Catalog, it is appended to file names automatically by the R scripts
