# Supplementary material for: Correction: Sinking towards destiny: High throughput measurement of phytoplankton sinking rates through time-resolved fluorescence plate spectroscopy
Source: PLoS One. 2018 Apr 24;13(4):e0196624. doi: 10.1371/journal.pone.0196624 (PMC5915776; doi:10.1371/journal.pone.0196624)
Supplement: S1 Script — (ZIP) [file pone.0196624.s001.zip › SinkWORX/ManualMaterial/SinkWorxManual.docx]

SinkWorx

*A system for high throughput measurement of phytoplankton sinking rates using a plate spectrofluorometer with downstream analysis in “R”*

https://www.dropbox.com/sh/w03c2nt97rjk990/AACyg_nJrl75ztT9s6A89zT-a?dl=0

Table of Contents

[Overview of SinkWorx 2](#_Toc492896253)

[*Advantages* 3](#_Toc492896254)

[*Disadvantages* 3](#_Toc492896255)

[PlateGrowthWorx Directory Structure 4](#_Toc492896256)

[Getting Started with SinkWorx 5](#_Toc492896257)

[Saving Your Fluorescence Data 8](#_Toc492896258)

[Common Issues and How to Solve Them 9](#_Toc492896259)

@2017

Catherine Bannon

Melissa Rioux

Douglas A. Campbell

Overview of SinkWorx

SinkWorx is a system for high throughput measurement of phytoplankton sinking rates using a plate spectrofluorometer with downstream analysis in R.

It is a series of R scripts housed in a directory, that uses a catalog of the cultures under study and specifically named spectrofluorometer files saved to specifically named folders. The system allows us to:

1. Import data saved from a plate spectrofluorometer.

(*SpectraWorks running a Molecular Devices Gemini EM, as implemented, but customizable to other instruments)*

1. Assemble the data into a vector of measurements for each well, on each plate, under analyses.
2. Plot the measurement vectors vs. time vectors that are assembled from the measurement time.
3. Fit the measurement vs. time plots with a curve fit to estimate the sinking rate of the cells in the culture.

*Advantages*

- Plate spectrofluorometers (or spectrophotometers) are widely available
- Many microbes grow well in well plates, allowing parallel tracking of phenomics or physiomics
- R is open source and is well supported by an ever-growing community of developers
- Allows for thousands of data points to be analyzed with only a few clicks

*Disadvantages*

- Maximum well volumes (6 to 96 well plate) are only ~0.5 to 6 ml, limiting some post-growth analyses
- Contamination across wells is a risk
- R has installation and learning curves
- File formats from plate spectrofluorometers can be tricky
- Maintaining the culture catalog and directory structures requires thought and ongoing attention

SinkWORX Directory Structure

SinkWORX is stored in a DropBox directory and includes:

i) SinkWORXManual (this file)

ii) SinkingCulture_Catalog.csv

A formatted .csv file in which you will list information about each culture (each well on each plate) under study.

iii) SinkingRates

A folder that in turn contains an example 'Project Folder'

Coscis2016_SinkingRates

That in turn contains date & time folder(s), ex.

2017.06.06 14.25

The date and time folder must be named in the exact format

2017.06.06 14.25

At each time you save a plate spectrofluorometer measurement file you will save it into a specific date & time folder.

iv) SinkWorx also contains the actual R scripts:

Linear Decay Model Script.R

Linear Model Fit by Well.R

Sinking Raw Data Import.R

SinkWorx uses the R library ("here") to help manage file paths.

Getting Started with SinkWorx

To use SinkWorx you create a local folder within your 'Documents' folder, to contain the PlateGrowthWorx files.

On Macs this should be the default; most PC users will need to change the directory location:


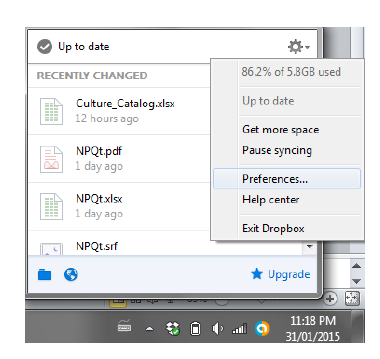

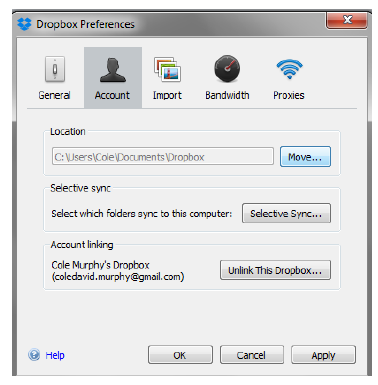


https://www.dropbox.com/sh/w03c2nt97rjk990/AACyg_nJrl75ztT9s6A89zT-a?dl=0

i) Entry the mandatory information in SinkingCulture_Catalog.csv

| Taxon | Time | Interval | Round | Well | Plate.Innoc.Date | Ex.WaveL | Em.WaveL | Growth.Temp | Project.Name | Mut.ID | CO2 | Growth.Light | Plate.Type |
| --- | --- | --- | --- | --- | --- | --- | --- | --- | --- | --- | --- | --- | --- |

ii) In the SinkingRates, create a project folder,

*e.g. "* Coscis2016_SinkingRates”

You will save all your growth data measurement files for the project within this folder.

iii) Measure the fluorescence emission at one or more wavelengths from each well of a well plate, using a Molecular Devices spectrofluorometer (e.g. Gemini EM) and the SoftMax software.

*The scripts can be adapted for other instruments, or for absorbance measurements.*

iv) Create a date & time folder, within the project folder (e.g. Coscis2016_SinkingRates) named in the format

"YYYY.MM.DD hh.mm"

e.g. 2017.06.06 14.25

*This name must be exactly formatted*

Saving Your Fluorescence Data

iv) Save each data .txt file with the following name format:

"Taxon.Growth Light.Plate.Round.txt", where:

- Taxon is the code for the organism you are growing, e.g. Syn or MED4;
- Growth Light is the numeric value of the growth light in µmol photons

m^-2^s^-1^; and

- Plate is the serial number of the plate used for this taxon, 1 – XX.
- Round is the experimental round for the plate being measured.

v) Put all .txt files from each measurement time in the date & time folder (YYYY.MM.DD hh.mm) within the project folder.

Common Issues and How to Solve Them

**i)** Improper entry of meta-data into SinkingCulture_Catalog.csv.

For example, two wells with identical meta-data may cause failures.

**ii)** If SinkingCulture_Catalog.csv is edited in a non-English system, or using non-English versions of Excel or other software the .csv separator may change.

**iii)** Typing or formatting inconsistencies in file or folder names.

**iv)** Moving among Mac, Windows & Linux platforms and versions may cause problems with file path formats for data import and results export, although the scripts attempt to be universal.

**v)** Molecular Devices (and other manufacturers?) change the data encoding of their export files over time and software versions.

You may need to change the 'file_encoding' setting in 'Master Growth Script.R'.

If necessary, see:

R Documentation; Functions to Manipulate Connections (Files, URLs, ...)

<https://support.rstudio.com/hc/en-us/articles/200532197-Character-Encoding>

**vi) PlateGrowthWorx Raw Data Import**

**Problem:** R is unable to access directories necessary to run the script

**Probable Causes and Solutions #1:**

1. There is a typo in a file path.
2. The SinkingCulture_Catalog has been left open, R cannot access a .csv currently being worked on. Simply make sure that these files are saved and closed prior to running the script.
3. Your version of R does not recognise the ~ function. You will need to remove the ~ and copy the file paths directly by right clicking on the directories and selecting “properties”. This problem seems to be more common on PCs than on Macs
4. Your computer is not connected to the internet. Simply check to make sure you are connected.

Reference

Bannon CC, Campbell DA (2017) Sinking towards destiny: High throughput measurement of phytoplankton sinking rates through time-resolved fluorescence plate spectroscopy. PLoSOne, doi 10.1371/journal.pone.0185166
